# Clinical relevance of copy number profiling in oral and oropharyngeal squamous cell carcinoma

**DOI:** 10.1002/cam4.499

**Published:** 2015-07-21

**Authors:** Pauline M W van Kempen, Rob Noorlag, Weibel W Braunius, Cathy B Moelans, Widad Rifi, Suvi Savola, Ronald Koole, Wilko Grolman, Robert J J van Es, Stefan M Willems

**Affiliations:** 1 Department of Otorhinolaryngology, University Medical Center UtrechtUtrecht, The Netherlands; 2 Department of Oral and Maxillofacial Surgery, University Medical Center UtrechtUtrecht, The Netherlands; 3 Department of Pathology, University Medical Center UtrechtUtrecht, The Netherlands; 4 Tumor diagnostics, MRC-HollandAmsterdam, The Netherlands

**Keywords:** 11q13, copy number aberrations, HPV, lymph node metastases, oral cavity SCC, oropharyngeal SCC

## Abstract

Current conventional treatment modalities in head and neck squamous cell carcinoma (HNSCC) are nonselective and have shown to cause serious side effects. Unraveling the molecular profiles of head and neck cancer may enable promising clinical applications that pave the road for personalized cancer treatment. We examined copy number status in 36 common oncogenes and tumor suppressor genes in a cohort of 191 oropharyngeal squamous cell carcinomas (OPSCC) and 164 oral cavity squamous cell carcinomas (OSCC) using multiplex ligation probe amplification. Copy number status was correlated with human papillomavirus (HPV) status in OPSCC, with occult lymph node status in OSCC and with patient survival. The 11q13 region showed gain or amplifications in 59% of HPV-negative OPSCC, whereas this amplification was almost absent in HPV-positive OPSCC. Additionally, in clinically lymph node-negative OSCC (Stage I–II), gain of the 11q13 region was significantly correlated with occult lymph node metastases with a negative predictive value of 81%. Multivariate survival analysis revealed a significantly decreased disease-free survival in both HPV-negative and HPV-positive OPSCC with a gain of Wnt-induced secreted protein-1. Gain of *CCND1* showed to be an independent predictor for worse survival in OSCC. These results show that copy number aberrations, mainly of the 11q13 region, may be important predictors and prognosticators which allow for stratifying patients for personalized treatment of HNSCC.

## Introduction

Head and neck squamous cell carcinoma (HNSCC) is the sixth most common cancer worldwide and is characterized by a biologically highly heterogeneous group of tumors. Treatment is predominantly based on location and TNM-classification and comprises mainly conventional methods such as surgery, radiotherapy, chemotherapy, or a combination of these. Currently, no treatment modalities exist that rely on the tumor-specific biology of HNSCC. The 5-year overall survival remains relatively poor at ∼50–60% and has not changed significantly over the past decades 1,2. Moreover, existing treatment modalities do not benefit patients equally and are often associated with (systemic) toxicities that reduce compliance and prevent timely completion of therapy 3. Molecular profiling is an essential step toward an increased understanding of the pathogenic biology of HNSCC. Such knowledge may help to optimize treatment efficacy, thereby improving locoregional control and survival among patients with HNSCC. Thus, the discovery of novel molecular biomarkers may pave the road for individualized cancer treatment 4,5.

Previous studies have shown an association between certain types of HNSCC and the presence of human papillomavirus (HPV). Molecular profiling may prove valuable to determine the exact role of HPV in oropharyngeal squamous cell carcinoma (OPSCC) and to the prediction of occult nodal metastasis in oral cavity squamous cell carcinoma (OSCC). Along with known risk factors such as alcohol and tobacco consumption, HPV infection has been identified to play an etiologic role in HNSCC, especially in OPSCC 6. Recent studies reveal molecular differences between HPV-positive and HPV-negative tumors. 7–11. Most HPV-positive OPSCCs seem to result in a favorable clinical outcome and show better response to radiotherapy compared to their HPV-negative counterparts 12. This suggests that HPV-positive OPSCCs could be treated with de-escalation protocols to minimize therapy-related side effect without compromising on treatment outcome. However, recent studies show that in a considerable portion of HPV-positive tumors worse clinical outcome has been observed 13. This subgroup of HPV-positive OPSCC should potentially be identified by means of molecular profiling to determine the need for additional treatment as opposed to treatment de-escalation. In early (Stage I–II) OSCC, reliable prediction of nodal metastasis is crucial for selecting appropriate treatment. Unfortunately, in 30–40% of these tumors even optimal imaging with MRI, CT, and ultrasound with aspiration cytology is insufficient to accurately detect nodal disease. New diagnostic tools such as molecular tumor profiling have shown promising results to improve the negative predictive value (NPV) and thus are valuable to future treatment planning 14,15.

Constituting an important element in the causal chain to cancer initiation and progression, genetic imbalances could serve as predictive or prognostic biomarkers in the near future. Genomic copy number aberrations (CNA) are alterations of the DNA resulting in an abnormal copy number of a region within the DNA. To find potential relevant aberrations for clinical decision making, this study correlates CNAs of a panel of 36 common oncogenes and tumor suppressor genes in two major subsites of HNSCC, knowingly the oral cavity and the oropharynx, with both clinicopathological features and survival.

## Materials and Methods

### Patient selection and clinicopathological information

The study population was described previously 10,16. In short, from the pathologic archives of the University Medical Center Utrecht, all cases of primary histologically proven OPSCC (1997–2011), and all small (clinically T1–2 classification) primary histologically proven OSCC (2004–2010) were selected. Demographical, clinical, and survival data were retrieved from electronic medical records.

For OPSCC and OSCC, respectively, material from biopsies and resection specimen was used. Since we used leftover tissue from routine diagnostic procedures, no ethical approval was required according to the Dutch national ethical guidelines (www.federa.org). Anonymous or coded use of leftover tissue for scientific purposes is part of the standard treatment agreement with patients in our center 17. Archived formalin-fixed paraffin-embedded primary OPSCC and OSCC specimens were used for multiplex ligation-dependent probe amplification (MLPA). From 383 tumors (202 OPSCC and 181 OSCC) enough tissue was available for suitable DNA extraction.

For all OSCC margin status, tumor diameter, thickness, and the histological features of the tumor front, that is, invasive pattern, perineural, and vascular invasive growth, were assessed by a dedicated head and neck pathologist (S. M. W.). In addition, specimens consisting of normal oral cavity mucosa of patients treated for an oral fibroma (due to chronic irritation by dentures or dental prosthesis) with no history of head and neck cancer were used as controls in OSCC experiments. Normal oropharynx mucosa biopsies derived from patients with neck metastases from an unknown primary tumor in head and neck region were used as controls in OPSCC experiments.

### HPV DNA detection

HPV type 16 positive tumors were determined by a validated test algorithm as described before 10. First, each paraffin-embedded oropharynx tumor was stained with an antibody against p16 (clone 16P07; Neomarkers, Fremont, CA). A case was considered positive when at least 70% of tumor cells showed strong nuclear and/or cytoplasmic staining 18. Tumors positive for p16 were subsequently analyzed using the Linear array HPV Genotyping test (S01710; Roche, Almere, the Netherlands) as well as the Linear array Detection kit (S03373; Roche) to confirm HPV-positive status. For quality control, HPV16 positive tonsil control tissue was used as positive control and normal skin tissue as negative control. Both were included in each run.

### DNA extraction

Hematoxylin and eosin-stained slides were reviewed by a dedicated head and neck pathologist (S. M. W.) to confirm the presence of malignancy. Samples with a tumor percentage of at least 30% were included in this study. After deparaffinization, corresponding tumor areas were scraped off from 5 *μ*m paraffin blank slides using a scalpel. Tumor tissue was suspended in direct lysis buffer (50 mmol/L Tris-HCl, PH 8.0; 0.5% Tween 20) and subsequently lysed by overnight incubation at 56°C in proteinase K (10 mg/mL; Roche, Almere, the Netherlands), followed by heat inactivation at 98°C for 10 min and subsequently DNA extraction by means of centrifugation after which the supernatant was recovered.

### Multiplex ligation-dependent probe amplification

After centrifugation, 5 *μ*L of isolated DNA was used for MLPA analysis according to the manufacturer's instructions. A set of 36 genes for 12 different chromosomal locations (Probe mix P428-B1 HNSCC; MRC Holland, Amsterdam, the Netherlands) was investigated. For this kit, the genes were selected based on a thorough literature search (by S. S. and W. R.) for frequent CNAs in HNSCC in association with prognosis. Table S1 shows the contents of this probemix and includes chromosomal locations of all probes. All tests were performed in duplicate in a professional thermocycler (Biometra, Goettingen, Germany). Seven references samples (five normal oropharynx or oral cavity tissues without CNA and two blood samples) were included in each MLPA experiment. Reaction products were separated by electrophoresis on an ABI 3730 capillary sequencer (Applied Biosystems, Foster City, California, USA). Gene copy numbers were analyzed using Genemapper software v4.1 (Applied Biosystems) and Coffalyser.NET analysis software (MRC Holland). For reliable performance of MLPA reactions, a minimum of 20 ng of sample DNA is recommended. MLPA quality was ascertained by means of three different procedures. First, the probemix contains Q-fragments, which can detect low DNA concentrations. Signaling from these Q-fragments is repressed by the MLPA-probes as long as a sufficient amount of DNA is used. If Q-fragments exceed one-third of the ligation-dependent control fragments, this indicates the sample contains too little DNA. In our study, such samples were excluded from further analysis 19. Second, 11 internal reference probes (chromosomal regions in which copy number alterations were not expected) are included in the probe mix P428-B1 HNSCC. If more than two reference probes were aberrant, test results were considered invalid. Third, if duplicates were inconsistent, the sample was excluded from further analysis. The analysis of copy number status using MLPA includes two steps: the comparison of the copy number ratios of the patient sample with the internal reference probes and, secondly the comparison of the copy number ratios of a patient sample with normal tissue (healthy tissue in which copy number for the reference probes and genes of interest are expected to be normal). Cutoff values were defined as before; an MLPA copy number ratio below 0.7 was defined as loss, 0.7–1.3 as normal, above 1.3 as gain, and values above 2.0 as amplification 20.

### Statistics

All statistical analyses were performed using IBM (Armonk, New York, USA) SPSS 20.0 statistical software. MLPA results were dichotomized as loss versus no loss (cut-off 0.70), gain versus no gain (cutoff 1.3), and nonamplified versus amplified (cut-off 2.0). The Pearson chi-square test (or Fisher's exact when appropriate) was used to compare baseline characteristics for categorical variables and frequencies of loss, gain, or amplification for individual genes and chromosomal arms between HPV-negative and HPV-positive OPSCC and between lymph node-positive and lymph node-negative OSCC. The analysis of variance test was used to compare continuous variables (e.g., age) between these groups in baseline characteristics. Backward logistic regression was performed to compare CNA between HPV-positive and HPV-negative OPSCC, taking into account differences in clinicopathological features between the two groups.

Disease-free survival (DFS) was used for survival analysis. DFS was defined as survival after primary treatment without any signs or symptoms of recurrent or persistent disease. Both recurrence and death were recorded as events. Since over 95% of all HNSCC recurrences occur within 36 months after treatment and patients in our center are discharged from follow-up after a disease-free period of 60 months, analysis was cutoff at 60 months. Univariate analysis was demonstrated by Kaplan–Meier curves and statistical significance was determined using log rank tests. Multivariate analysis was performed using the Cox proportional hazard model. Clinicopathological characteristics both significantly related to survival as well as those acting as possible confounders (as determined by Cox regression analysis) were included in the multivariate model. The level of significance was set at *P*-value <0.05.

## Results

### Copy number analysis of OPSCC and OSCC: descriptive analysis

In 28 cases, the quality or quantity of DNA was insufficient for MLPA analysis which resulted in the availability of copy number data for 355/383 (92%) tumors (191 OPSCC, 164 OSCC) from our initial study population. Twenty-one percent (41 out of 191) of the OPSCCs were positive for high-risk HPV. All HPV-positive tumors contained HPV type 16, whereas two of 41 were co-infected with HPV 33 or HPV 52 as well. None of the OSCC were positive for high-risk HPV. Clinicopathological features of our study population are listed in Table1.

**Table 1:** Patient and tumor characteristics

Characteristic	Oral cavity (%)	Oropharynx (%)
Sex		
Male	98 (60)	134 (70)
Female	66 (40)	57 (30)
Age		
Mean, range (years)	61, 23–90	59, 35–88
Smoking		
No	81 (49)	47 (25)
Yes	83 (51)	144 (75)
Alcohol		
No	76 (46)	65 (34)
Yes	88 (54)	126 (66)
Clinical AJCC tumor size		
T1	80 (49)	18 (9)
T2	84 (51)	55 (29)
T3	0 (0)	41 (21)
T4	0 (0)	77 (40)
Clinical AJCC nodal status		
N0	144 (88)	47 (25)
N1–3	20 (12)	144 (75)
Histological AJCC nodal status		
N0	109 (66)	N.A.
N1–3	55 (34)	
Stage (based on clinical TNM)		
I	72 (44)	5 (3)
II	72 (44)	20 (10)
III	14 (9)	27 (14)
IV	6 (4)	139 (73)
High risk HPV		
No	164 (100)	150 (79)
Yes	0 (0)	41 (21)
Extra capsular spread^1^		
No	153 (93)	N.A.
Yes	11 (7)	
Infiltration depth^1^		
≤4 mm	50 (30)	N.A.
>4 mm	114 (70)	
Vascular invasive growth^1^		
No	149 (91)	N.A.
Yes	15 (9)	
Perineural growth^1^		
No	115 (70)	N.A.
Yes	49 (30)	
Noncohesive tumor front^1^		
No	53 (33)	N.A.
Yes	110 (67)	
Missing	1	

AJCC, American Joint Committee on Cancer; HPV, human papillomavirus; N.A., not applicable.

1 Histological variables were only available from the oral cavity.

### CNA and HPV status in OPSCC

**Figure 1:**
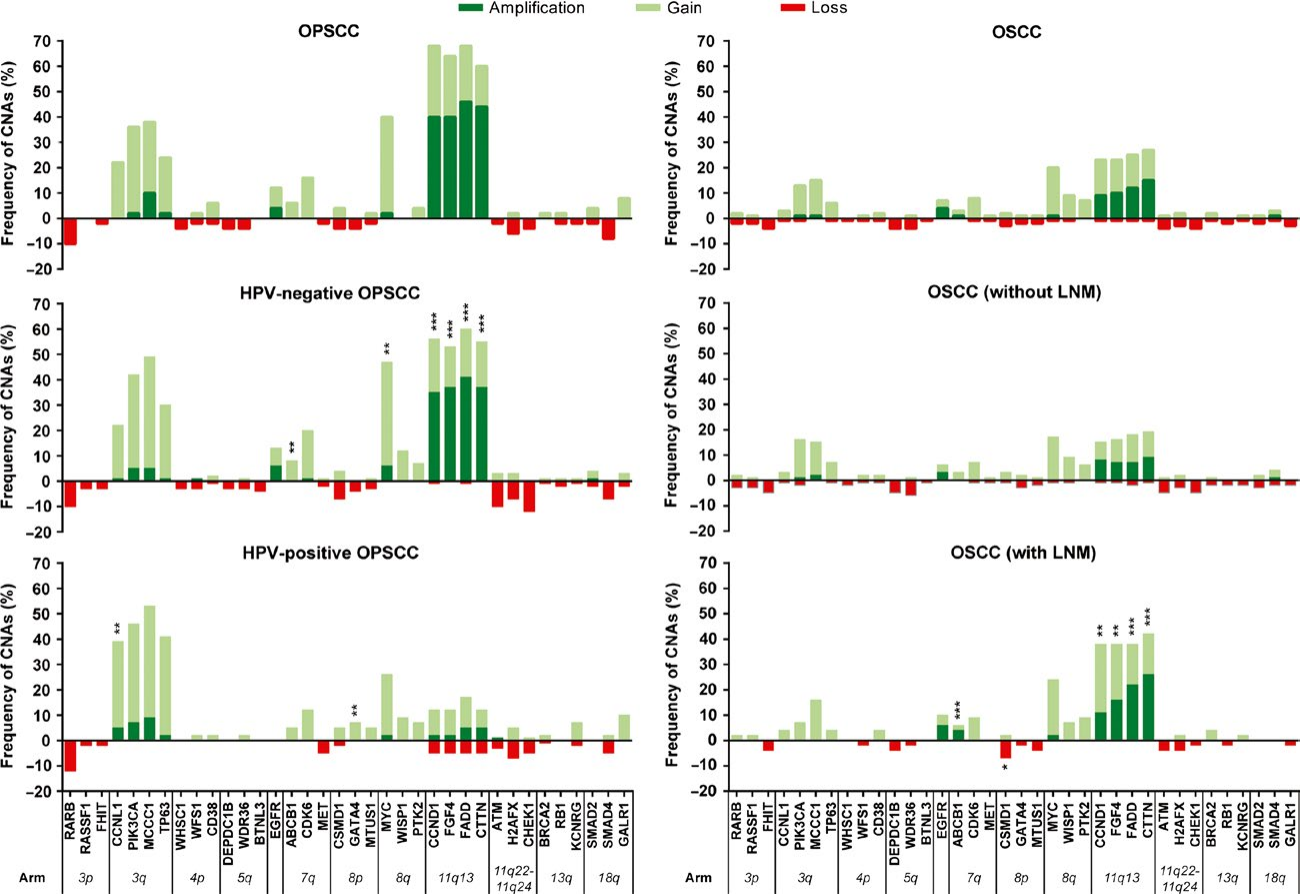
Frequencies of copy number aberrations. Comparison of frequency of copy number aberrations in their genomic order between Left: HPV-positive versus HPV-negative OPSCC and Right: lymph node-positive versus lymph node-negative OSCC. Significant differences in *loss, **gain, ***gain, and amplification. OPSCC, oropharynx squamous cell carcinoma; OSCC, oral cavity squamous cell carcinoma; HPV, human papillomavirus.

Forty-one (21%) patients with an OPSCC showed an HPV-positive tumor. At baseline, patients with HPV-positive tumors had a significantly lower alcohol intake and smoked less than patients with HPV-negative tumors. Patients with HPV-negative tumors presented with larger tumors and were clinically less suspected to have lymph node metastases (LNM) (Table S2). Differences between gene copy number status of the 36 analyzed genes in HPV-negative and HPV-positive OPSCCs are presented in Figure1 (left). CNAs were found in 157 cases (82%). HPV-negative tumors showed a significantly (*P* = 0.04) higher total number of CNAs compared to HPV-positive tumors. Gain of *CCNL1* was independently associated with positive HPV status. Copy number gain of *EGFR* and both amplification and gain of genes located at 11q13 (*FADD*, *CTTN*, *CCND1*, and *FGF4*) were significantly more frequent in HPV-negative tumors. After correction for baseline differences, multivariate analyses revealed that *FADD*, *CTTN*, *CCND1*, and *FGF4* were independently associated with negative HPV status. No significant differences in frequencies of gene copy number losses were observed between HPV-positive and HPV-negative OPSCCs. Besides these observed differences, several genes showed frequent aberrations in both HPV-positive and HPV-negative OPSCC. The genes *CCNL1*, *PIK3CA*, *TP63*, *MYC*, *MCCC1*, and *CDK6* showed a recurrent gain (>10% of cases) and *RARB* showed a recurrent loss (>10% of cases) in each group.

### CNA and nodal metastasis in early OSCC

CNA of the 36 analyzed genes in lymph node-positive and negative OSCC are illustrated in Figure1 (right). In the whole cohort of 164 OSCCs, gain and amplification (chromosomal region 11q13, *CCND1*, *FGF4*, *FADD*, and *CTTN*) and loss (*CSMD1*) correlated significantly with LNMs. However, in the clinically relevant subgroup of clinically lymph node-negative OSCC (Stage I–II, *n* = 144), statistical significance of amplification in several of these biomarkers disappeared. In this clinically relevant subgroup, gain of chromosomal region 11q13 had the most diagnostic value for determining occult LNM (*P* = 0.002) with a NPV of 81% (95% confidence interval [CI] 72–89%), see Table2. The genes *MCCC1* and *MYC* were commonly gained (>10%) in OSCC with and without LNMs.

**Table 2:** Copy number aberrations in early OSCC correlated with LNM

Gene/arm	All stages (164 tumors)		*P*-value^1^	Clinical Stage I–II (144 tumors)		*P*-value^1^
	pN0	pN1–3		pN0	pN1–3	
Gain/amplification						
*CCND1*						
Normal	92 (73)	34 (27)	0.001	90 (79)	24 (21)	0.013
Gain	8 (35)	15 (65)	NS	8 (47)	9 (53)	NS
Gain and amplification	9 (60)	6 (40)		9 (69)	4 (31)	
*FGF4*						
Normal	91 (73)	34 (27)	0.002	89 (79)	24 (21)	0.019
Gain	10 (46)	12 (54)	NS	10 (56)	8 (44)	NS
Gain and amplification	8 (47)	9 (53)		8 (61)	5 (39)	
*FADD*						
Normal	89 (72)	34 (28)	0.006	87 (80)	22 (20)	0.008
Gain	12 (57)	9 (43)	0.007	12 (60)	8 (40)	NS
Gain and amplification	8 (40)	12 (60)		8 (53)	7 (47)	
*CTTN*						
Normal	88 (73)	32 (27)	0.002	86 (80)	21 (20)	0.005
Gain	11 (55)	9 (45)	0.005	11 (61)	7 (39)	0.045
Gain and amplification	10 (42)	14 (58)		10 (53)	9 (47)	
*11q13*						
Normal	90 (74)	32 (26)	0.001	88 (81)	21 (19)	0.002
Gain	10 (46)	12 (54)	0.030	10 (50)	10 (50)	NS
Gain and amplification	9 (45)	11 (55)		9 (60)	6 (40)	
Loss						
*CSMD1*						
No	108 (68)	51 (32)	0.044	106 (76)	33 (24)	0.016
Loss	1 (20)	4 (80)		1 (20)	4 (80)	

Percentage values are given in parenthesis. OSCC, oral cavity squamous cell carcinomas; LNM, lymph node metastases; pN0, histological lymph node negative; pN1–3, histological lymph node positive; NS, not significant.

1 For gain/amplification data, upper *P*-value represents chi-square test of gain versus normal and lower *P*-value represents chi-square test of amplification versus no amplification.

### Survival analysis

**Figure 2:**
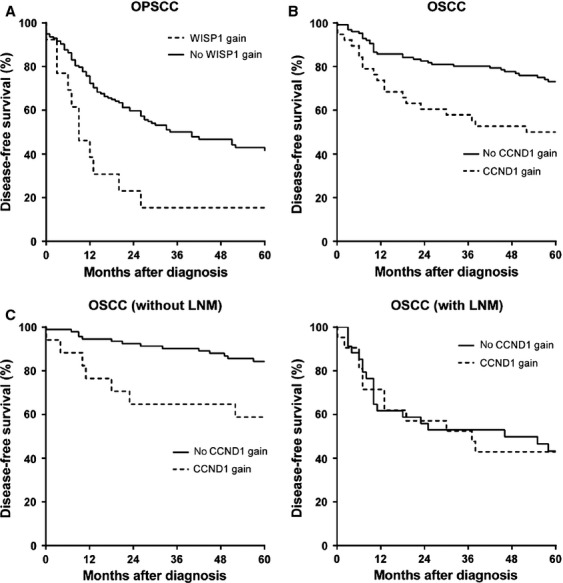
Kaplan–Meier curves of disease-free survival. (A) OPSCC: log rank *P* = 0.003, hazard ratio = 2.48 (1.32–4.68) *P* = 0.005. (B) clinical T1–2 OSCC: log rank *P* = 0.003, hazard ratio = 2.28 (1.30–4.02) *P* = 0.004. (C) clinical T1–2 OSCC: without LNM log rank *P* = 0.008, hazard ratio = 3.21 (1.30–7.97) *P* = 0.012; with LNM log rank *P* = 0.909, hazard ratio = 0.91 (0.51–2.15) *P* = 0.910. OPSCC, oropharynx squamous cell carcinoma; OSCC, oral cavity squamous cell carcinoma; LNM, lymph node metastases.

For survival analysis, only patients treated with curative intention were included. Hereto, 22 cases of OPSCC were excluded from survival analysis. DFS was defined as survival without recurrence of disease. The mean DFS oncologic follow-up of patients alive without recurrence was 40 months for OPSCC and 58 months for OSCC. In OPSCC, the baseline characteristics age, clinical nodal metastases (N1–3), clinical advanced T classification (T3–T4), and HPV negativity were significantly correlated with a decreased DFS. From the 36-gene panel, amplification of *FADD* and gain of wnt-induced secreted protein-1 (*WISP1*) correlated with a worse DFS. Multivariate analysis was performed to estimate the association of all analyzed factors with DFS. Gain of *WISP1*, age, advanced T stage (T3–T4), clinical nodal metastasis, and HPV-negative status were correlated independently with decreased DFS in OPSCC, see Figure2 and Table3.

**Table 3:** Multivariate analysis DFS in oral and oropharyngeal SCC

	Characteristic	Hazard ratio (95% CI)	*P*-value
Oropharyngeal SCC	*WISP1* gain	2.63 (1.34–5.15)	0.005
	Age	1.04 (1.01–1.07)	0.002
	Clinical T3–4	1.92 (1.18–3.13)	0.008
	Clinical N1–3	2.19 (1.25–3.84)	0.006
	HPV negativity	2.66 (1.44–4.93)	0.002
Oral SCC (only pN0)	*CCND1* gain	3.07 (1.24–7.63)	0.016
	Age	1.07 (1.02–1.12)	0.003

DFS, disease-free survival; SCC, squamous cell carcinoma; HPV, human papillomavirus.

In OSCC, both gain and amplification of chromosomal region of 11q13 and its individual genes (*CCND1*, *FGF4*, *FADD*, *CTTN*) correlated with a decreased DFS, with *CCND1* gain acting as the strongest predictor (hazard ratio 2.28 with 95% CI 1.28–4.02, *P* = 0.004). Multivariate survival analysis revealed a different effect of *CCND1* gain on DFS pending on nodal status: in lymph node-positive tumors no correlation between gain and DFS was found, while lymph node-negative tumors with *CCND1* gain had a significantly worse survival than lymph node-negative tumors without *CCND1* gain, see Figure2. Besides age, *CCND1* gain was an independent predictor for worse DFS in this subgroup of OSCC, see Table3.

## Discussion

Gene CNA play a key role in cancer development and progression and thus are of prognostic as well as therapeutic value in clinical cancer care 21. In this retrospective study, the copy number status of 36 head and neck cancer-associated genes was examined in 355 patients with primary OSCCs or OPSCCs and coupled to clinically relevant features such as HPV status in OPSCC, occult LNM in early stage OSCC, and patient survival. To our knowledge, this study constitutes the largest cohort of oral and oropharyngeal cancers with known HPV status, CNAs, and survival described so far.

### HPV status in OPSCC

In our OPSCC cohort, 21% of the tumors were high-risk HPV-positive, which is in line with earlier reports of high-risk HPV prevalence in the Netherlands 18. Within the group of OPSCC, there were significant copy number differences between HPV-positive and HPV-negative OPSCC. Gain and amplification of four genes located on 11q13 (*FADD*, *CTTN*, *FGF4*, and *CCND1*) and gain of *EFGR* occurred more frequently in the HPV-negative tumors. The relationship between CNA and HPV status in OPSCC has been shown in five previous studies 7,8,22–24. However, the sample size of these studies was rather small and only one study investigated the correlation between genetic aberrations and patient survival 8. Our findings are in line with previous studies showing that HPV-negative tumors display significantly more amplifications as well as genetic aberrations in total 7,8,22,24. This could be explained by inactivation of p53 and the retinoblastoma protein due to viral oncoproteins E6 and E7 in HPV-positive OPSCC, whereby the number of required genetic aberrations for carcinogenesis is lower in these tumors compared to HPV-negative tumors 25,26. The genes *FADD*, *CTTN*, *FGF4*, and *CCND1* are all located at chromosomal region 11q13. This region is the most frequently amplified region in HNSCC and is associated with unfavorable prognosis 27. In our study, 38% of HPV-negative tumors showed 11q13 amplification compared to only 2% in HPV-positive tumors. As this is consistent with other studies, it strongly associates HPV-negative tumors with 11q13 amplification 8,24. One gene located at 3q region, *CCNL1*, was significantly associated with HPV-presence, as stated in one previous study 22. However, this study contained only 25 tonsillar carcinomas and gain of the 3q region in total (mean of four tested genes located at this region) was not related to HPV presence in our study. Furthermore, our findings support results from two previous studies identifying 3q gain as the most frequently observed aberration in HPV-positive as well as HPV-negative tumors 7,8.

### LNM in OSCC

In early OSCC, appropriate management of the neck region is still topic of debate. Current strategies include elective neck dissection (END), sentinel node biopsy (SNB), irradiation, and watchful waiting. According to the decision tree analysis developed by Weis et al. in 1994, management that consists of observation only—as opposed to END—is an accepted treatment modality if the probability of occult LNM is less than 20% 28. Recent publications recommend thresholds between 17% and 44% 29,30. However, these thresholds are not very reliable as the quality of the evidence is limited 31. During the last decade, more studies have focused on the diagnostic value of the SNB, mainly because of its association with lower morbidity compared to END. SNB has an overall NPV of ∼95% in early OSCC and a slightly lower NPV of about 88% in floor of mouth tumors 32,33. Unfortunately, SNB is an invasive technique requiring general anesthesia and surgery which may hinder a subsequent neck dissection and is related to complications in patients with specific comorbidities. As a consequence, noninvasive diagnostic biomarkers for occult LNM with a NPV above 80% are of clinical relevance for treatment decision making. Our study shows that both amplification and gain of 11q13 (or its individual genes) is correlated with occult LNM in clinically Stage I–II OSCC. In this CNA, panel of 36 oncogenes and tumor suppressor genes, gain or amplification (all ratios >1.3) instead of normal copy number of 11q13 is the most accurate biomarker, with an NPV of 81% and a positive predictive value of 46%. Twelve other studies correlated gain/amplification of 11q13, or its individual genes, with LNMs with various results. Six studies found a significant correlation between 11q13 amplification and LNM, but the other six found no correlation at all 34–45. In addition, pooled results of the five studies investigating *CCND1* amplification showed significant correlation (odds ratio 2.12, 95% CI 1.43–3.16, *P* < 0.001) with LNM 46. However, only one study investigated the diagnostic value of *CCND1* amplification in Stage I–II OSCC with an NPV of 83%, which is similar to our results 43. Possible explanations for our lack of correlation between *CCND1* amplification and LNM are the differences in the used detection method and cutoff values for amplification between these studies. Myo et al. used fluorescent in situ hybridization with ≥3 spots in >20% of 100 cells as cutoffs for amplification, which could be a less hard definition for amplification than a copy number change of >2 with MLPA in our study 43. Another possibility is sampling error. Although all samples included contained more than 30% tumor cells, due to tumor heterogeneity amplifications in a portion of OSCC could be insufficient to reach the amplification threshold of 2.0 with MLPA. This could also explain why gain and amplification of 11q13 both are indicative of more occult LNM.

### Survival

The exact prognostic value of *FADD* amplification in OPSCC is not clear. In our study, 11q13 amplification showed to predict worse survival in univariate analysis. However, this predictive ability was confounded by the strong correlation between *FADD* amplification and HPV status; in a multivariate model *FADD* amplification did not appear to be an independent predictor. Furthermore, in a subgroup of HPV-negative OPSCC 11q13 amplification or gain showed no association with outcome, altogether suggesting that 11q13 copy number gain or amplification has no prognostic value in OPSCC. Only one other study similarly found 11q13 amplification in OPSCC to be associated with worse overall survival 8. However, no multivariate analyses were performed to control for baseline differences and confounders.

Interestingly, gain of *WISP1* at 8q24.22 turned out to be a predictor for worse DFS in OPSCC, independent of HPV status. *WISP1* is a member of the CCN family (CYR61, CTGF, NOV), which is a group of six secreted proteins that regulates adhesion and migration or functions as growth factors that modulate cell proliferation and differentiation 47. Additionally, there is increasing evidence that WISP1 is involved in carcinogenesis 48. In esophageal squamous cell carcinoma, protein expression of WISP1 was found to be an independent prognostic factor for worse overall survival 49. A recent functional study confirmed that WISP1 mediates resistance to radiotherapy in esophageal squamous cancer 50. This implicates that *WISP1* could also play an important role in the development of HNSCC and might predict a poorer prognosis. Moreover, because of the possible role of *WISP1* in the development of radiation resistance, it is questionable to enroll HPV-positive tumors with *WISP1* gain in de-escalating trials.

Survival analysis in OSCC revealed gain of *CCND1* as a predictor for worse DFS. The correlation between *CCND1* gene aberrations and worse survival has been shown in other studies, though only Hanken et al. and Miyamoto et al. performed multivariate analyses 34,39,42–45. Miyamoto et al. found similar results, with nodal status and *CCND1* amplification being independent predictors for survival 44. On the other hand, *CCND1* amplification did not function as an independent predictor for survival in the study by Hanken et al. 34. These inconsistent results could be due to differences in the used definition for amplification (a gene/cell ratio >2.0 in Hanken et al. vs. ≥3 spots in >20% of 100 cells in Miyamoto et al.).

Additionally, *CCND1* gain has no prognostic value in patients with proven histologic LNM in our cohort of OSCC, however it does correlate with worse overall survival in patients without LNM. The DFS of patients with *CCND1* gain without LNM is comparable to patients with LNM, see Figure2. There are several possible explanations for this remarkable finding. First of all, in patients in the group of *CCND1* gain without proven LNM, micrometastases could have been present which are known to be potentially missed by pathologists examining a neck dissection specimen 51,52. Another possible explanation is the common function of the simultaneously amplified genes of 11q13 (*CTTN*, *FADD*, *CCND1*, and *FGF4*) in tumor growth and invasion (Table S1). This common function could account for a worse survival in patients without LNM. Tumors without gain of *CCND1*, but with LNM obviously have other molecular aberrations which make invasion and metastasis possible. This could account for the similar survival in cases of LNMs, regardless of *CCND1* gain.

### Limitations

This study was performed in a large consecutive cohort of OSCC and OPSCC patients. Nevertheless, some limitations require mentioning. First, although the OSCC data are derived from a prospective consecutive cohort, the OPSCC cohort has been gathered retrospectively and is nonconsecutive. Second, due to a limited registration of treatment response after radiotherapy DFS was used as a marker for treatment outcome in OPSCC. Therefore, it was not possible to correlate *WISP1* gain with response to radiotherapy. Although the correlation between *WISP1* gain and worse DFS could be explained by resistance to radiotherapy, these results should be validated in a prospective cohort with adequate treatment response follow-up. Third, we acknowledge that all used OSCC tissues are derived from resection specimens. To be of real clinical value to the prediction of occult nodal metastasis, these results similarly require validation in incisional biopsies from OSCCs. Finally, due to large differences in both intoxications (smoking and alcohol) and staging of OSCC and OPSCC, no reliable comparison of CNA between these sites could be made. Although there seem to be differences between these sites, see Figure2, this should be confirmed in a study with a more homogeneous set of oral and oropharyngeal cancers.

In conclusion, we have identified CNA that are associated with HPV status in OPSCC and with prognosis in OSCC. Furthermore, we showed that *WISP1* gain correlates with decreased DFS in OPSCC independent of HPV status, potentially due to radiotherapy resistance. These findings could have implications for de-escalation trials in HPV-positive OPSCC. Finally, we showed that 11q13 gain is a promising biomarker for predicting occult LNM in patients with clinically Stage I–II OSCC. Consequently, CNA profiling increases our understanding of the specific biology of HNSCC and may prove of considerable value to personalizing future cancer therapy in these patients.

## Supporting Information

**Table S1.** Contents of the HNSCC MLPA kit P428-B1. **Table S2.** Characteristics of 191 OPSCC by HPV status.
