# Supplementary material for: Clinical relevance of copy number profiling in oral and oropharyngeal squamous cell carcinoma
Source: Cancer Med. 2015 Jul 21;4(10):1525–35. doi: 10.1002/cam4.499 (PMC4618623; doi:10.1002/cam4.499)
Supplement: Supplementary file 1 [file cam40004-1525-sd1.docx]

| **Table S1. Contents of the HNSCC MLPA kit P428—B1** | | | | | |
| --- | --- | --- | --- | --- | --- |
| *Gene* | *Chromosome* | *Mapview position* | *Function related with carcinogenesis* | *Hallmark[*[*53*](#_ENREF_53)*]* | |
| ***Loss of 3p arm***  *Deletions of 3p arm are observed in ~60% of the HNSCC patient samples. Several target genes have been reported like FHIT (Mao L. et al. 1996 Cancer Res. 56:5128-31; Virgilio L. et al. 1996, PNAS, 93:9770-5; Gonzales MV. et al. 1998, J Clin Pathol. 51:520-4), RASSF1 (Hogg RP. et al. 2002 Eur J Cancer. 38:1585-92), and RARB (Zou CP. et al. 2001 Oncogene 20:6820-7).* | | | | | |
| *RARB* | 3p24.2 | 03-025.444279 | Inhibits cell growth | Evading growth suppressors | |
| *RASSF1* | 3p21.31 | 03-050,343037 | RAS-pathway regulation | Sustaining proliferative signaling | |
| *FHIT* | 3p14.2 | 03-059.974839 | Apoptosis and cell cycle regulation | Resisting cell death | |
| ***Gain of 3q arm***  *Gain of 3q has been associated with lymph node metastasis and poor prognosis in HNSCC (Bockmuhl U. et al. 2000, Am J Pathol. 157:369- 75; Ashman JNE et al. 2003, Br J Cancer. 89:864-9). Several candidate genes have been suggested including CCNL1 (Redon R. et al. 2002, Cancer Res, 62:6211-7; Sticht C. et al. 2005, Br J Cancer. 92:770-4), PIK3CA (Woenckhaus J. et al. 2002, J Pathol. 198:335-42), and MCCC1 (Jarvinen AK et al. 2008, Genes Chromosomes Cancer. 47:500-9), and TP63 (Hibi K. et al. 2000, PNAS, 97:5462-7; Muzio LL. et al. 2005, Hum Pathol. 36:187-94).* | | | | | |
| *CCNL** | 3q25.31 | 03-158.348937  03-158.359308 | Regulates G0–G1 cell-cycle progression | Evading growth suppressors | |
| *PIK3CA* | 3q26.32 | 03-180.410106 | Cell growth, proliferation and survival | Evading growth suppressors | |
| *MCCC1* | 3q27.1 | 03.184.252624 | Mostly unknown, function as catalyzer in mitochondria | Deregulating cellular energetics | |
| *TP63* | 3q28 | 03-190.832006 | Pro-apoptotic | Resisting cell death | |
| ***Loss of 4p14-pter***  *Deletion detected in ~40% of HNSCC patient samples (Ashman JNE et al. 2003, Br J Cancer, 89: 864–869).* | | | | | |
| *WHSC1* | 4p16.3 | 04-001.950156 | Regulation of genes with function in histone modification | Genome instability & mutation | |
| *WFS1* | 4p16.1 | 04-006.355788 | Mostly unknown, associated with endoplasmatic reticulum trafficking | Deregulating cellular energetics | |
| *CD38* | 4p15.32 | 04-015.389226 | Role in cell adhesion, signal transduction and calcium signaling. | Activating invasion & metastasis | |
| ***Loss of 5q***  *Deletion of 5q23-qter is detected in ~40% of HNSCC patients (Ashman JNE et al. 2003, Br J Cancer, 89: 864–869).* | | | | | |
| *DEPDC1B* | 5q12.1 | 05-060.018734 | DNA damage response | Genome instability & mutation | |
| *WDR36* | 5q22.1 | 05-110.467455 | Involved in a variety of cellular processes, including cell cycle progression, signal transduction, apoptosis, and gene regulation | Evading growth suppressors | |
| *BTNL3* | 5q35.3 | 05-180.365094 | Cell proliferation and development | Sustaining proliferative signaling | |
| ***Gain of 7p11.2***  *EGFR amplification is found in ~30% of HNSCC and it coincides with overexpression and poor survival of HNSCC patients (Sheu JJ. et al.2009, Cancer Res, 69:2568-76).* | | | | | |
| *EGFR** | 7p11.2 | 07-055.191962  07-055.236919 | Receptor tyrosine kinase involved in signal transduction | Sustaining proliferative signaling | |
| ***Gain of 7q***  *Increased MET expression associates with invasive HNSCC (Galeazzi E. et al. 1997, Eur Arch Otorhinolaryngol. 254:S138-43). 65% of HNSCC show gain of MET and 13% show amplification of MET gene (Speicher MR. et al. 1995, Cancer Res. 55:1010-3; Seiwert T. et al. 2009, Cancer Res. 69:3021-31).* | | | | | |
| *ABCB1* | 7q21.12 | 07-087.012074 | Cellular cholesterol regulation / release of mitochondrial cell death-promoting molecules | Resisting cell death | |
| *CDK6* | 7q21.2 | 07-092.085391 | Cell cycle control protein for G1 phase progression and G1/S transition | Evading growth suppressors | |
| *MET* | 7q31.2 | 07-116.197031 | Receptor tyrosine kinase involved in signal transduction | Activating invasion & metastasis | |
| ***Loss of 8p arm***  *Deletions of the whole or part of chromosome 8p arm are one of the most common cytogenetic abnormalities and loss of 8p has been reported in between 10 and 53% in HNSCCs. Loss of 8p23 is reported be an independent factor for poor prognosis in HNSCC (Bockmuhl U. et al. 2001, Otolaryngol Head Neck Surg. 124:451-5). Several target genes have been suggested including CSMD1 (Sun PC. et al. 2001, Genomics. 75:17-25), GATA4 (Lin L. et al. 2000, Cancer Res. 60:1341-7), and MTUS1 (Ye H. et al. 2007, Cancer Genet Cytogenet. 176:100-6).* | | | | | |
| *CSMD1* | 8p23.2 | 08-004.839277 | Proliferation | Sustaining proliferative signaling | |
| *GATA4* | 8p23.1 | 08-011.650003 | Cell survival by regulating the  expression of anti-apoptotic proteins | Resisting cell death | |
| *MTUS*** | 8p22 | 08-017.645396  08-017.702395  08-017.656483 | Cell differentiation and growth inhibiting | Evading growth suppressors | |
| ***Gain of 8q24***  *45-56% of HNSCC cases have gain or amplification of 8q (Squire JA. et al. 2002. Head Neck. 24:874-87). MYC, WISP1 and PTK2 have been suggested to be the target genes (Rodrigo JP. et al. 1996, Arch. Otolaryngol Head Neck Surg. 122:504-7; Agochiya M. et al. 1999, Oncogene. 18:5646-53; Jarvinen AK. et al. 2008, Genes Chromosomes Cancer. 47:500-9).* | | | | | |
| *MYC* | 8q24.21 | 08-128.817870 | Transcription factor involved in apoptosis and cell proliferation | Resisting cell death | |
| *WISP1* | 8q24.22 | 08-134.309095 | Enhanced cell survival by inhibitions of p53 mediated apoptosis | Resisting cell death | |
| *PTK2* | 8q24.3 | 08-141.879785 | Receptor tyrosine kinase involved in signal transduction of cell growth | Sustaining proliferative signaling | |
| ***Gain of 11q13***  *30-50% of HNSCC have gain of 11q13 (including CCND1, FGF4, FADD and CTTN (aka. EMSI)) and it seems to associate with larger tumor size, presence of lymph node metastasis, poor histological differentiation, advanced clinical stage and poor prognosis (Schuuring E. et al. 1992, Oncogene. 7:355-61; Muller D et al. 1994, Eur J Cancer B Oral Oncol. 30B:113-20; Xia J. et al. 2007, Oral Oncol. 43:508-14; Gibcus JH. et al. 2007, Clin Cancer Res. 13:6257-66).* | | | | | |
| *CCND1* | 11q13.3 | 11-069.171946 | Cell cycle control protein involved in signal transduction | Evading growth suppressors | |
| *FGF4* | 11q13.3 | 11-069.297353 | Involved in tumor growth and invasion | Activating invasion & metastasis | |
| *FADD** | 11q13.3 | 11-069.730527  11-069.727339 | Regulating cell proliferation and enhancing invasion, pro-apoptotic | Sustaining proliferative signaling | |
| *CTTN* | 11q13.3 | 11-069.956859 | Enhance cellular motility and play a role in tumor invasion | Activating invasion & metastasis | |
| ***Loss of 11q22-qter***  *11q22-qter is detected in 30-50% of HNSCC samples and 11q loss is associated with reduced sensitivity to ionizing radiation (Parikh RA. et al. 2007, Genes Chromosomes Cancer. 46:761-75).* | | | | | |
| *ATM* | 11q22.3 | 11-107.655436 | DNA damage sensor | Genome instability & mutation | |
| *H2AFX* | 11q23.3 | 11-118.471495 | DNA repair | Genome instability & mutation | |
| *CHEK1* | 11q24.2 | 11-125.018925 | Checkpoint mediated cell cycle arrest | Evading growth suppressors | |
| ***Loss of 13q***  *Loss of 13q occurs in more than 50% of primary HNSCCs and it is associated with poor prognosis (Li X. et al. 1994, J Natl Cancer Inst. 86(20):1524-9; Sabbir MG. et al. 2006, Int J Exp Pathol. 87:151-61).* | | | | | |
| *BRCA2* | 13q13.1 | 13-031.869059 | DNA-repair | Genome instability & mutation | |
| *RB1* | 13q14.2 | 13-047.937195 | Negative regulator of cell cycle | Evading growth suppressors | |
| *KCNRG* | 13q14.2 | 13-049.492724 | Pro-apoptotic and cell growth inhibition | Resisting cell death | |
| ***Loss of 18q***  *Loss of 18q is detected in 41-59% of HNSCC cases, and it associates with advanced stage and poor prognosis (Takebayashi S. et al. 2004, Genes, Chromosomes Cancer. 41:145-54). Several target genes of this loss have been suggested including SMAD4 (Bornstein S. et al. 2009, J Clin Invest. 119:3408-19), GALR1 (Kanazawa T. et al. 2007, Oncogene. 26:5762-71; Misawa K. et al. 2008, Clin Cancer Res, 14:7604-13) and SMAD2 (Mangone FR. et al. 2010, Mol Cancer. 9:106).* | | | | | |
| *SMAD2* | 18q21.1 | 18-043.628917 | The signal of the transforming growth factor (TGF)-beta, and thus regulates multiple cellular processes, such as cell proliferation, apoptosis, and differentiation. | Sustaining proliferative signaling | |
| *SMAD4* | 18q21.2 | 18-046.838518 | Involved in many cell functions such as differentiation, apoptosis, gastrulation, embryonic development and the cell cycle. | Sustaining proliferative signaling | |
| *GALR1* | 18q23 | 18-073.109588 | Growth regulatory function | Evading growth suppressors | |
| *Reference probes* | | | | | |
| *DPYD* | 1p21.3 | 01-097.688408 |  | |  |
| *PEX13* | 2p16.1 | 02-061.126370 |  | |  |
| *RPIA* | 2p11.2 | 02-088.779111 |  | |  |
| *LRRFIP1* | 2q37.3 | 02-238.337227 |  | |  |
| *PKHD1* | 6p12.3 | 06-051.858618 |  | |  |
| *NOS1* | 12q24.22 | 12-116.253160 |  | |  |
| *POMT2* | 14q24.3 | 14-076.842475 |  | |  |
| *SPG11* | 15q21.1 | 15-042.648954 |  | |  |
| *PRPF31* | 19q13.42 | 19-059.323257 |  | |  |
| *USP25* | 21q21.1 | 21-016.172591 |  | |  |
| *PPIL2* | 22q11.21 | 22-020.379682 |  | |  |
| * For these genes, probes for two different regions are present.  ** For this gene, probes for three different regions are present. Because of bad correlation between the second probe and the other two probes we excluded this probe in further analyses. | | | | | |

| **Table S2. Characteristics of 191 OPSCC by HPV status** | | | |
| --- | --- | --- | --- |
| Patient or tumor characteristics | HPV-positive (%) | HPV-negative (%) | p-value |
| No. of cases | 41 (21) | 150 (79) | - |
| Age  (Average (range) | 58 (35-80) | 60 (40-88) | 0.321 |
| Sex  Male  Female | 32 (78)  9 (22) | 102 (68)  48 (32) | 0.213 |
| Smoking history  Never or quit >1 year  Yes or quit < 1 year | 21 (51)  20 (49) | 26 (17)  124 (83) | < 0.001 |
| Alcohol use  Never or quit > 1 year  Yes or quit < 1 year | 21 (51)  20 (49) | 44 (29)  106 (71) | 0.009 |
| Overall AJCC stage  Stage I-II  Stage III-IV | 4 (10)  37(90) | 22 (15)  128 (85) | 0.416 |
| AJCC tumor size*  T1-2  T3-4 | 21 (52)  19 (48) | 51 (34)  99 (66) | 0.032 |
| AJCC nodal stage**  N0  N1-3 | 4 (10)  37 (90) | 41 (30)  106 (70) | 0.016 |
| Treatment  RT/ RT+ Chemo  S/S+RT/S+RT+Chemotherapy  None | 32 (78)  8 (20)  1 (2) | 122 (81)  22 (15)  6 (4) | 0.691 |
| Second primary tumors  Negative  Positive | 40 (98)  1 (2) | 132 (80)  18 (12) | 0.070 |
| * 1 missing ** 3 missing values  *Abbreviations*: HPV, human papillomavirus; RT, Radiotherapy; S, surgery; AJCC, American Joint Committee on cancer | | | |

**Reference**

53. Hanahan, D., and R. A. Weinberg. 2011. Hallmarks of cancer: the next generation. Cell 144:646–674.
